# *Pseudomonas oryzihabitans* sepsis in a 1-year-old child with multiple skin rashes: a case report

**DOI:** 10.1186/s13256-017-1230-6

**Published:** 2017-03-23

**Authors:** Michael Owusu, Ellis Owusu-Dabo, Godfred Acheampong, Isaac Osei, John Amuasi, Nimako Sarpong, Augustina Annan, Hsin-Ying Chiang, Chih-Horng Kuo, Se Eun Park, Florian Marks, Yaw Adu-Sarkodie

**Affiliations:** 1 Kumasi Centre for Collaborative Research in Tropical Medicine, Kwame Nkrumah University of Science and Technology, Kumasi, Ghana; 2 Department of Global Health, School of Public Health, Kwame Nkrumah University of Science and Technology, Kumasi, Ghana; 3 Agogo Presbyterian Hospital, PO Box 27, Agogo, Ashanti Region Ghana; 4 Institute of Plant and Microbial Biology, Academia Sinica, 128 Section 2, Academia Road, Nankang, Taipei 11529 Taiwan; 5 Department of Epidemiology, International Vaccine Institute, SNU Research Park, 1 Gwanak-ro, Gwanak-gu, Seoul, 08826 South Korea; 6 Department of Clinical Microbiology, Kwame Nkrumah University of Science and Technology, Kumasi, Ghana

**Keywords:** *Pseudomonas oryzihabitans*, Case report, Skin infections, Sepsis, *16S* ribosomal DNA

## Abstract

**Background:**

*Pseudomonas oryzihabitans* is a *Pseudomonas* bacterial organism rarely implicated in human infections. The bacterium has been isolated in a few reported cases of neurosurgical infections and patients with end-stage cirrhosis, sickle cell disease, and community-acquired urinary tract infections. Limited information exists in developing countries, however, because of the lack of advanced microbiological tools for identification and characterization of this bacterium. This case report describes the isolation of a rare *Pseudomonas* bacterium in a patient presenting with sepsis and skin infection.

**Case presentation:**

A 1-year-old girl was presented to a hospital in the northeastern part of Ghana with a 1-week history of pustular rashes on her scalp and neck, which occasionally ruptured, along with discharge of yellowish purulent fluid. The child is of Mole-Dagbon ethnicity and hails from the northern part of Ghana. *Pseudomonas oryzihabitans* was identified in the patient’s blood culture using the 16S ribosomal deoxyribonucleic acid sequencing technique. The rash on the patient’s scalp and skin resolved after continuous treatment with gentamicin while her condition improved clinically.

**Conclusions:**

This finding suggests the potential of this bacterium to cause disease in unsuspected situations and emphasizes the need to have evidence for the use of the appropriate antibiotic in clinical settings, particularly in rural settings in Africa. It also brings to the fore the unreliability of conventional methods for identification of *Pseudomonas* bacteria in clinical samples and thus supports the use of 16S ribosomal deoxyribonucleic acid in making the diagnosis.

## Background

*Pseudomonas oryzihabitans* is the current name of a non-lactose-fermenting bacterium that used to be called *Chromobacterium typhiflavum* and *Flavimonas oryzihabitans* [1]. The species name *typhiflavum* was originally described by Dressel and Stickl as a variant of typhoid bacillus [2]. The bacterium is gram-negative and oxidase-negative with typical yellow-pigmented colonies visualized on agar media. It is thought to survive in moist environments, especially rice paddies, although some researchers have cultured cases from inhalational therapy equipment and hospital sinks [3, 4]. The organism has rarely been implicated as pathogenic in humans and often is not given serious attention in clinical practice.

Since the 1970s, when the first case of bacteremia in a neurosurgical patient with extradural cerebral hemorrhage secondary to *P. oryzihabitans* was reported, other researchers have identified the pathogen in patients with cancer, end-stage cirrhosis, sickle cell disease, and community-acquired urinary tract infections [5, 6]. *P. oryzihabitans,* unlike the other forms of *Pseudomonas* bacteria (for example, *Pseudomonas aeruginosa*, and *Burkholderia cepacia*) is susceptible to quinolones, carbapenems, β-lactamase inhibitor drugs, and trimethoprim/sulfamethoxazole [4, 7]. Treatment algorithms for managing *P. oryzihabitans* are not available in Ghana and many other developing countries, particularly those in sub-Saharan Africa. We report a case that is of significance because of the rare occurrence of this pathogen, the unique microbial characteristics of the bacteria, the age of the child involved, and the consequential clinical outcome.

## Case presentation

A 1-year-old girl was presented to a hospital in the northeastern part of Ghana with a 1-week history of pustular rashes on her scalp and neck, which occasionally ruptured, along with discharge of yellowish purulent fluid. The child is of Mole-Dagbon ethnicity and hails from the northern part of Ghana. The patient had also experienced recurrent episodes of fever for 3 days, which had been controlled with the use of acetaminophen. The child had no known history of human immunodeficiency virus (HIV) infection, tuberculosis, or other underlying medical condition and lived with her parents, whose primary occupation was farming. The child lives in a rural area about 20 km from the hospital. On examination, the patient weighed 8 kg, was pale and febrile with a temperature of 39.7 °C, and well-hydrated. Her heart rate was 132 beats per minute, and her heart sounds were normal. Her lungs were clear, and her respiratory rate was 35 breaths per minute. A provisional diagnosis of sepsis and malaria was made.

Pending the results of laboratory tests of collected blood, urine, and stool samples, the patient was empirically treated with 270 mg of intravenous cefuroxime three times daily and 40 mg of intravenous gentamicin daily for 48 hours. Intravenous artesunate 24 mg was also administered for the first 24 hours (Table 1).

**Table 1:** Timeline of treatment

Parameters	Day 1	Day 2	Day 3	Day 4	Day 5	Day 6	Day 7
Total white cell count	8.8 × 10^9^/L	Not done	8.5 × 10^9^/L	10.8 × 10^9^/L	Not done	Not done	9.4 × 10^9^/L
Hemoglobin	6.6 g/dl	Not done	5.1 g/dl	12.2 g/dl	Not done	Not done	10.6 g/dl
Antibiotics	Intravenous cefuroxime 270 mg three times daily						
Antibiotics	Intravenous gentamicin 40 mg daily						
Antibiotics					Flucloxacillin suspension 5 ml four times daily		
Antimalarial	Artesunate 24 mg/24 hours						
Malaria rapid diagnostic test	Positive						
Gram stain of broth	Positive (gram-negative rods)						
Blood culture isolate			Positive				
Hemotransfusion			240 ml/4 hours				

Complete blood count investigations showed a hemoglobin concentration of 6.6 g/dl and a total white blood cell count of 8800 cells/μl (lymphocytes 52.1%, neutrophils 37.4%). The result of a malaria rapid diagnostic test was positive for malaria. The patient was given a hemotransfusion of 240 ml of whole blood over a period of 4 hours.

Results of both urine and stool cultures were negative for any bacterial growth. The blood culture sample was flagged as positive after 24 hours of incubation in an automated BD BACTEC 9050 blood culture system (BD Biosciences, San Jose, CA, USA), and a Gram stain of the culture broth revealed gram-negative rods. The broth was subcultured on blood agar, chocolate agar, and MacConkey agar (Oxoid Ltd, Basingstoke, UK) and incubated at 37 °C and 30 °C. Growth characteristics at 37 °C were poor, with grayish nonhemolytic colonies on blood agar and nonlactose fermentation on MacConkey agar. Plates incubated at 30 °C showed good pure growth on MacConkey agar and nonhemolytic colonies on blood agar. Results of biochemical investigations were negative for oxidase and urease and positive for citrate. The isolate was subjected to analytical profile index (API) 20NE and 20E (bioMérieux, Marcy-l’Étoile, France), which yielded unacceptable results.

Molecular identification was performed by extracting deoxyribonucleic acid (DNA) from a pure culture of this bacterium using SpheroLyse DNA extraction kit (Hain Lifescience GmbH, Nehren, Germany) according to the manufacturer’s instructions. The 16S ribosomal DNA (rDNA) was amplified using the primer pair 8F and 1492R [8]. The polymerase chain reaction conditions and the subsequent steps of Sanger sequencing we used were based on those described by Liu *et al*. [9]. The resulting 16S rDNA sequence was checked using DECIPHER (version 2.2.0; R Foundation for Statistical Computing, Vienna, Austria) to verify that it was not a chimera [10].

The procedure for molecular phylogenetic inference was based on that described by Ku *et al*. [11]. Briefly, the 16S rDNA sequence of the isolated strain was used as the query for sequence similarity search against the GenBank 16S rDNA sequence database. Highly similar sequences representing closely related species were selected for phylogenetic inference. All sequences were aligned using Multiple Sequence Comparison by Log-Expectation (MUSCLE) [12], and the resulting multiple sequence alignment was used to infer a maximum likelihood phylogeny using PhyML (version 3.0) [13]. The bootstrap support values were inferred based on resampling 1000 iterations.

**Fig. 1:**
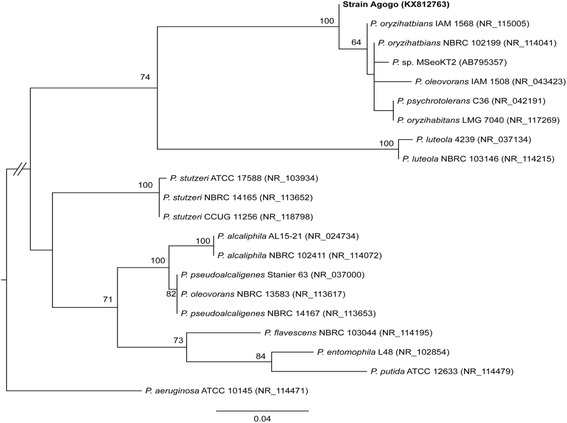
Maximum likelihood molecular phylogeny based on 16S ribosomal DNA sequences. The GenBank accession numbers are provided in parentheses after the strain names. The newly isolated strain *Agogo* reported in this study is highlighted in *bold*. The levels of bootstrap support are labeled above internal branches; only values greater than 60% are listed

The isolated strain reported in this study was found to be most similar to *Pseudomonas oryzihabitans* IAM 158 [GenBank accession NR_115005] with nucleotide sequence identity of 1455/1460 (99.7%). The molecular phylogeny also indicated that the new sequence is clustered with other *P. oryzihabitans* strains, which strongly supports the findings displayed in Fig. 1. The purified bacterial strain has been assigned the strain name *Agogo* to reflect its geographic origin and the sequence deposited in the GenBank database [accession number KX812763].

Antimicrobial susceptibility testing was done on the isolated bacteria using the Kirby-Bauer disk diffusion method following Clinical Laboratory Standards Institute guidelines [14]. The isolate was found to be susceptible to co-trimoxazole, chloramphenicol, ciprofloxacin, and gentamicin but resistant to ampicillin and cefuroxime. The rash on the patient’s scalp and skin, as well as her temperature spikes, resolved after continuous treatment with gentamicin, and her condition improved clinically. She was discharged on flucloxacillin and folic acid therapy 1 week after admission. A final diagnosis of septicemia secondary to bacterial skin infection with malaria coinfection was made.

## Discussion

*P. oryzihabitans* is a rare bacterial isolate that has been linked to meningitis, endocarditis, diabetes mellitus, and infection with other organisms, especially in immunocompromised individuals [4, 15]. The pathogenicity of *P. oryzihabitans* is still a subject of debate in the scientific community, hence the need for adequate information to enable clinicians and public health practitioners to manage episodes associated with this pathogen.

Although it might be difficult to determine to what extent the severity of our patient’s illness was due to malaria or sepsis, the presence of the rare pathogen *P. oryzihabitans* in the blood in our patient is of importance, being the first such case reported from West Africa and only the second in sub-Saharan Africa [15]. It is possible that the pustular rashes provided an entry route for the bacteria directly from the environment, and the patient eventually progressed to sepsis. It is also possible that the child might have been exposed through skin bites or minor abrasions, either in the house or upon visiting her family’s farm. Our patient’s case is similar to one reported in South Africa, which occurred in a 4.7-year-old child who had been diagnosed with kwashiorkor, sepsis, and anemia [16]. Six cases of skin and soft tissue infections caused by *P. oryzihabitans* have been reported in patients with a previous histories such as surgery, immunosuppression, diabetes mellitus, trauma, bites, and lactation [17]. Although we did not perform an immunological immunodeficiency test for our patient, the result of a screening test for HIV antibody was negative, thus ruling out possible immunodeficiency disease associated with her illness.

An important feature we observed was the unique microbiological characteristics of this bacterium and the failure of the API to accurately identify the pathogen. The biochemical properties of our isolate using the Analytical Profile Index Non-Enterobacteriaceae (API NE) differed from the usual reported cases of *P. oryzihabitans* by being positive for gelatin hydrolysis and capric acid assimilation and negative for mannose and maltose assimilation [18]. Bosshard *et al*. [19] observed the low sensitivity of the API 20NE and thus recommended the use of 16S ribosomal ribonucleic acid (RNA) gene sequencing as an effective means of identification of clinically relevant nonfermenting gram-negative bacilli.

## Conclusions

Our report shows that a combination of conventional and molecular technologies is the best approach to identifying Pseudomonas bacteria which may be implicated in the clinical condition of this child.
